# HSP70 mRNA expression by cells of the epithelial rest of Malassez due to mechanical forces in vitro

**DOI:** 10.1186/s12903-016-0181-4

**Published:** 2016-02-18

**Authors:** Hideki Kogai, Kei Nakajima, Tungalag Ser-Od, Akram Al-Wahabi, Kenichi Matsuzaka, Taneaki Nakagawa, Takashi Inoue

**Affiliations:** Department of Clinical Pathophysiology, Tokyo Dental College, 2-9-18, Misaki-cho, Chiyoda-ku Tokyo, 101-0061 Japan; Sanno Hospital, Minato-ku, Tokyo, 107-0052 Japan; Oral Health Science Center, Tokyo Dental College, Chiyoda-ku Tokyo, 101-0061 Japan; Department of Dentistry and Oral Surgery, Keio University School of Medicine, Shinjuku-ku Tokyo, 160-8582 Japan

**Keywords:** HSP70, Epithelial rest of Malassez, Mechanical force, in vitro, Centrifugation

## Abstract

**Background:**

The purpose of the present study was to examine the in vitro responses of ERM cells under the combination of centrifugal and compression forces, in terms of their expression of HSP70 mRNA.

**Methods:**

The ERM cells were positive for CK19 indicating that they were derived from the odontogenic epithelium. Cultured ERM cells were applied centrifugal force and compressing force at one to three times as mechanical forces. After addition of forces, cells were observed using scanning electron microscope (SEM) and were measured expression of HSP70 mRNA by RT-PCR.

**Results:**

SEM observations showed the cells were flattened immediately after the application of mechanical force, but nuclear protrusions recovered the same as the control 3 h later. A significantly higher expression of HSP70 mRNA was observed in ERM cells under mechanical force compared with the control, but it gradually decreased with time. No accumulation of HSP70 mRNA expression occurred with intermittent force. However, the expression of HSP70 mRNA with intermittent force repeated 3 times was significantly higher compared with intermittent force applied only once or twice.

**Conclusions:**

These findings suggest that ERM cells express HSP70 mRNA in response to mechanical force, and that intermittent force maintains the level of HSP70 mRNA expression.

## Background

It is known that the space of the periodontal ligament (PDL) is maintained throughout life. Some studies have reported that the epithelial rest of Malassez (ERM) most probably contributes to the maintenance of the PDL width [1]. The ERM is separated from Hertwig’s epithelial root sheath (HERS) at the embryonic stage of tooth root development. HERS cells are tightly connected and are surrounded by a continuous basement membrane. When dental papilla cells are attached to the HERS, the inner basement membrane of the HERS is intermittent immediately after those cells start to synthesize a dentin matrix. Dental sac cells then migrate between the fragmented HERS [2]. After the cementum synthesized by these mesenchymal cells of the dental sac, epithelial cells aggregate to form cell clusters named the ERM, which are again surrounded by a continuous basement membrane and are usually located near the cementum area in the PDL or in the cementum after the eruption of teeth, and are not related to aging [3]. Carrie and Katchburian reported that apoptosis of ERM cells may be part of the mechanism of turnover of ERM [4]. Many studies have reported both the morphological and functional changes of ERM cells under various conditions in vivo [5–7]. Inoue et al. reported that regeneration occurred when an alveolar bone-PDL-tooth cavity was prepared, however, dento-alveolar ankylosis occurred 2 months after the operation. They observed that no ERM exists in the regenerated PDL and they concluded that the absence of the ERM must be related with dento-alveolar ankylosis [5]. Furthermore, Yamashiro et al. reported that denervation of the inferior alveolar nerve leads to a reduced distribution of the ERM at 1 week and dento-alveolar ankylosis occurs at 6 weeks. They also confirmed that ERM receptor was immune-positive for trkA, which shows a high-affinity to the NGF receptor and they concluded that the sensory nerve might play a regulatory role in maintaining the ERM [6]. Mine et al. observed the healing process of the PDL after tooth re-plantation in rats and compared the occluded group with the non-occluded group. They found that dento-alveolar ankylosis was clearly detected in the non-occluded group, but was not detected in the occluded group. They concluded that occlusal stimuli promote the regeneration of the PDL and prevent dento-alveolar ankylosis [7]. These facts suggest that the reduction of ERM distribution in the PDL might precede the development of dento-alveolar ankylosis and that mechanical stimuli must prevent dento-alveolar ankylosis. However, only a few studies have been reported about changes of the ERM under the various kinds of mechanical forces in vitro [8].

In general, the stress response is considered to represent a cellular defense mechanism against environmental disturbances [9–12]. If cells are exposed either to a mild or a moderate stress that is sufficient to up regulate the expression of heat shock proteins (HSPs), they are often able to survive subsequent, otherwise lethal stress stimuli. HSPs can be expressed by all types of cells and they play a protective role against a variety of harmful factors, including oxidants, inflammation, hypoxia, hyperthermia and also mechanical stimuli include orthodontic force [10–14]. Furthermore, strong orthodontic force induced apoptosis of PDL fibroblasts, and many ERM cells also fell into apoptosis [15, 16]. It was confirmed that HSP70 which serves in maintaining homeostasis was acted as a cochaperone by HSP40, can inhibit apoptosis by interfering with the function of apoptosis inducing factor (AIF) [17]. The effect of HSP70 on apoptotic protease-activating facor 1 (Apaf-1) probably accounts for its ability to provide resistance to the reported stress-induced apoptosis and its expression exists in the PDL throughout life [18].

The purpose of the present study was to examine the responses of ERM cells under mechanical forces in vitro, in terms of the expression of mRNA encoding HSP70, which is a stress resistance protein.

## Methods

### Cell culture

Porcine ERM cells were donated by Prof. Yoshihiro Abiko (Oral Pathology of Hokkaido Medical Science University). The ERM cells were cultured in minimum essential medium (MEM) supplemented with 10 % fetal bovine serum (FBS) and gentamycin in 75 mm culture dishes in a humidified atmosphere of 95 % air and 5 % CO_2_ at 37 °C. After sub-culture 3 times, ERM cells were inoculated in 35 mm dishes. When cells had been cultured for 7 days and reached 70 % confluence, they were used for the experiments.

### Exploratory experiment: determination of mechanical force in vitro (Fig. 1)

**Fig. 1:**
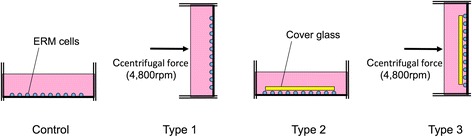
Illustration shows the application methods of mechanical force. For the centrifugation, the rotor rotates at 4,800 rpm for 20 min to load the mechanical force (Type 1). For the compressing force, a cover glass, 24 x 24 mm^2^, 0.2 g in weight, was placed on the cell surface for 20 min (Type 2). Combination of Type 1 and Type 2 (Type 3)

Type 1: Centrifugal force (4,800 rpm) for 20 min (*n* = 4)

Type 2: Compressing force (20 min) (*n* = 4)

Type 3: Compressing force + Centrifugal force (4,800 rpm) for 20 min (*n* = 4)

For centrifugal force, horizontal microplate rotor in a Hitachi centrifuge (HimacCT6D®) were used. The centrifugal experiment referred to by Redlish et al. establishes a pressure model by centrifugation of PDL cells in vitro [19, 20]. After the 35 mm culture dishes were inserted in the rotor adaptor, the device was set at a centrifugal force of 4,800 rpm (47.2 kPa, 482 g/cm^2^), which is nearly the same as a rapid expansion force [13].

For compressing force, a cover glass, 24 x 24 mm^2^ in size and 0.2 g in weight, was directly put on the cell surface (Fig. 1). There have been no report about centrifugal force with compression materials. Hoshina et al. used 9.0 g glass plate, however, cells under the glass plate with centrifugal force resulted death [14]. Then we tried to find proper compressive force under 0.2 g cover glass.

For the combination of compression and centrifugal force, a cover glass (24 x 24 mm^2^, 0.2 g) was directly placed on the cells and was removed after centrifugation at 4,800 rpm.

Cells in each of 3 types above were continuously cultured for 12 h and without any force was used as control.

**Fig. 2:**
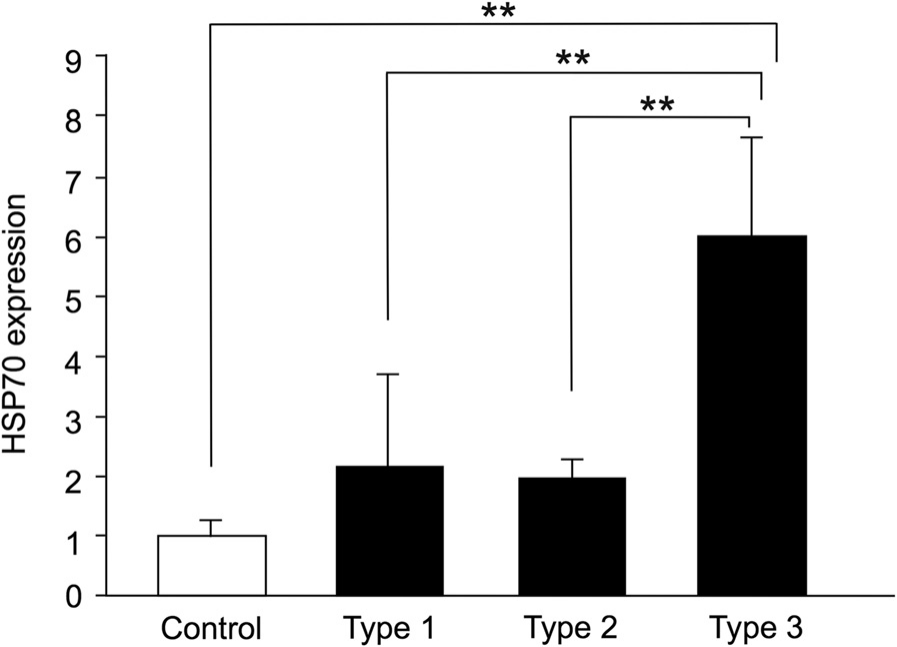
HSP70 mRNA expression at 3 h after different mechanical stimuli. Type 3 shows a significantly higher expression of HSP70 mRNA than the control, as well as type 1 and cover type 2. There was no significant difference among the control, type 1 and type 2, although both type 1 and type 2 tended to be higher than the control group. **p < 0.01

As a results, type 3 showed a significantly higher expression of HSP70 mRNA than the control, type1 and type2 (Fig. 2). Thus we decided to use the combined centrifugation and cover glass group as the experimental group.

### Scanning electron microscopic (SEM) observations

ERM cells were observed before, immediately after and 3 h after mechanical force using an Elionix ERA-8900FE field emission electron beam 3D surface roughness analyzer (4Channel 3 dimensional scanning electron microscope (4Ch-3D-SEM, Elionix Co., Ltd., Tokyo, Japan) at an accelerating voltage of 15 kV. For SEM observations, cells were initially fixed with 2 % glutaraldehyde for 1 h at 4 °C. The cells were then dehydrated in a graded series of ethanol, dried by tetramethylsilane (Merck KGaA, Darmstadt, Germany) and then sputter-coated with Au-Pd (Bio-Rad, Tokyo, Japan). The height of the cells was measured as a one-line cut system on the counter line figure using Image J analysis software (NIH, Bethesda, MD, USA).

### Experimental design

#### Experiment 1

ERM cells were collected using cell scraper after the addition of mechanical force at 3 h, 6 h, 12 h, 24 h and 36 h, and the expression of HSP70 mRNA was measured. ERM cells without any forces were used as control.

#### Experiment 2

Experiment 2 investigated whether the number of intermittent mechanical forces influences the accumulation of HSP70 mRNA (Table 1). The cover glass was removed after the centrifugation and the culture was continued until the next additional force.

**Table 1:** Experiment design of experiment 2

Time	Group 1	Group 2	Group 3
0	-	-	-
3 h	-	-	Force
6 h	-	Force	Force
9 h	Force	Force	Force
12 h	mRNA	mRNA	mRNA

Force, Addition of centrifugal force; mRNA, Isolation of RNA; −, No force

Group 1: Nine h after the culture, a centrifugal force (4,800 rpm) was applied for 20 min and the cells were observed 3 h after continuous culture.

Group 2: Six h after the culture, a 1^st^ centrifugal force (4,800 rpm) was applied for 20 min and 3 h after the continuous culture, a 2^nd^ centrifugal force (4,800 rpm) was applied for 20 min and the cells were observed 3 h after continuous culture.

Group 3: Three h after the culture, a 1^st^ centrifugal force (4,800 rpm) was applied for 20 min and 3 h after the continuous culture, a 2^nd^ centrifugal force (4,800 rpm) was applied for 20 min and 3 h after the continuous culture, a 3^rd^ centrifugal force (4,800 rpm) was applied for 20 min and cells were observed 3 h after the continuous culture (Table 1).

### Real-time reverse transcription PCR (RT-PCR)

Total RNA was extracted using the acid guanidium thiocyante/phenochloroform method, using a total RNA isolation reagent (Trizol Reagent, Invitrogen,USA) according to the manufacturer’s instructions. The RNAs were reverse-transcribed to cDNAs in a Thermal Cycler using Oligo (dT) primer (Invtirogen), RNase Inhibitor (Takara), Reverse transcriptase (Takara), dNTP Mixture and RNA PCR buffer (Sigma). A quantitative RT-PCR assay was carried out with a LightCycler™ (Roche Diagnostics, Mannheim, Germany) using the double-stranded DNA dye SYBR Green 1 (Roche Diagnostics) in order to observe the level of mRNAs. Primer sequence and size used in this study were shown in Table 2. Quantification was performed by comparing the levels obtained with standard samples as a previous study [21]. In the present study, the concentrations of cDNA in the unstimulated samples were 0.2, 0.5, 1.0 and 2.0 μl. Melting curve analyses were performed after the PCR amplification and to confirm no primer dimmer in the PCR products. The ratios of HSP70 mRNA expression were adjusted by the value of the housekeeping gene GAPDH.

**Table 2:** Primer sequence and size used in this study

Gene	Sequence		Size
HSP70	Forward	5’-CGGACGAGTACAAGGTTGA-3’	206
	Reverse	5’- CTCTTTCTCCGCCAACTG-3’	
GAPDH	Forward	5’-AGGGGCTCTCCAGAACATCA-3’	196
	Reverse	5’-GCCTGCTTCACCACCTTCTT-3’	

### Statistical analysis

Data are presented as means ± SD. The significance of differences was established by ANOVA followed by Scheffe’s tests using Excel 2013 (Microsoft, Redmond, WA, USA). Differences are considered significant at *p* < 0.01.

## Results

### SEM observations

**Fig. 3:**
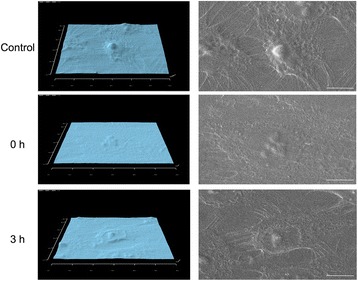
SEM observations. The rounded nuclei and flattened cytoplasm of cultured ERM cells were observed in the control group. Only flattened cells without any nuclear protrusions were observed immediately after the centrifugation and few protrusions of nuclei were observed at 3 h after the centrifugation. Scale bar, 10 μm

The ERM cells were spread out and nucleus protrusions and flattened cytoplasm were evident in the control group. The nucleus protrusions became flatter immediately after the centrifugation and nuclear protrusions were again observed at 3 h after the centrifugation (Fig. 3).

**Fig. 4:**
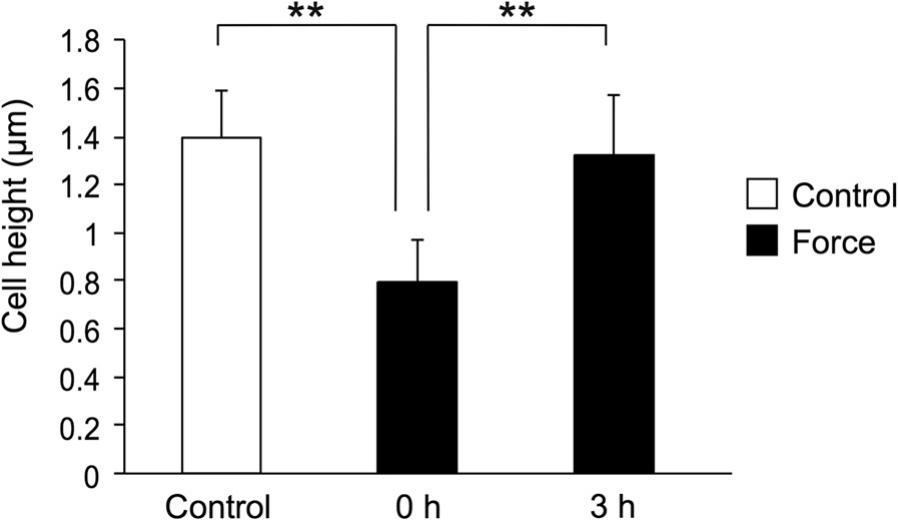
Cell height after the centrifugation. The height of ERM cells right after centrifugation was significantly lower than both the control and at 3 h after centrifugation. **p < 0.01

The average height of ERM cells was 1.4 ± 0.2 μm in the control group. The height of the ERM cells was 0.78 ± 0.18 μm immediately after the centrifugation and it recovered to the control level at 3 h after the centrifugation. The height of ERM cells immediately after the centrifugation was significantly lower than both the control and at 3 h after the centrifugation (Fig. 4).

### Expression of HSP70 mRNA by mechanical forces

#### Experiment 1

**Fig. 5:**
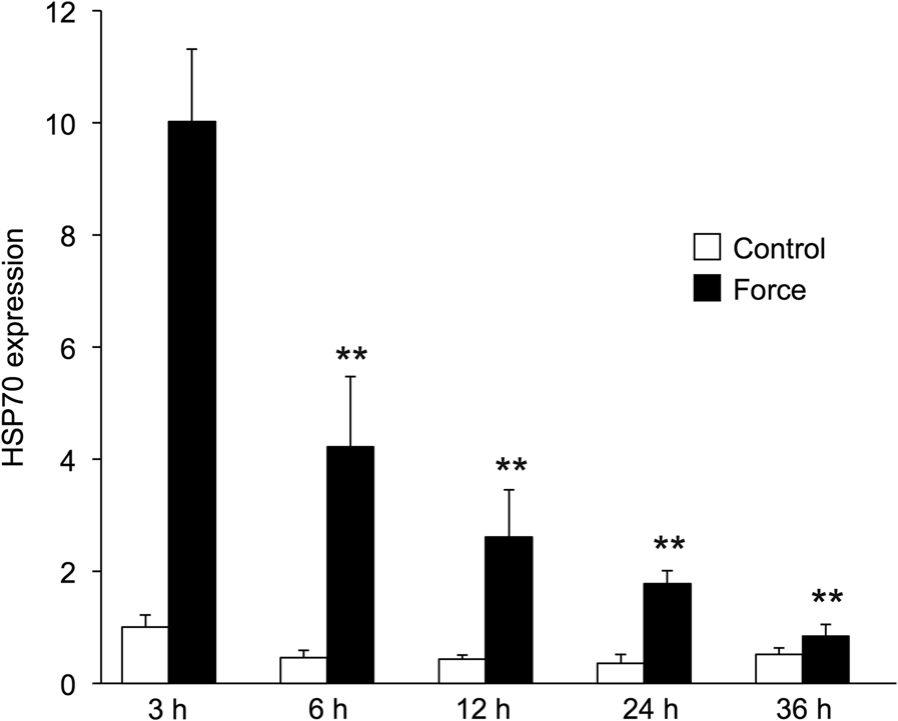
HSP70 mRNA expression at 3 h, 6 h, 12 h, 24 h and 36 h after the mechanical force. The expression of HSP70 mRNA at 3 h after the centrifugation was the highest and the expression gradually decreased until 36 h. The mRNA expression level did not remain constant and disappeared with time. **p < 0.01, compared to 3 h after the mechanical force

The expression of HSP70 mRNA by the experimental group (centrifugation and compression) was significantly higher than the control group at all time periods examined (Fig. 5). The expression of HSP70 mRNA of the experimental group was highest at 3 h after adding the force. The expression level of HSP70 mRNA was not stable and decreased with time.

#### Experiment 2

**Fig. 6:**
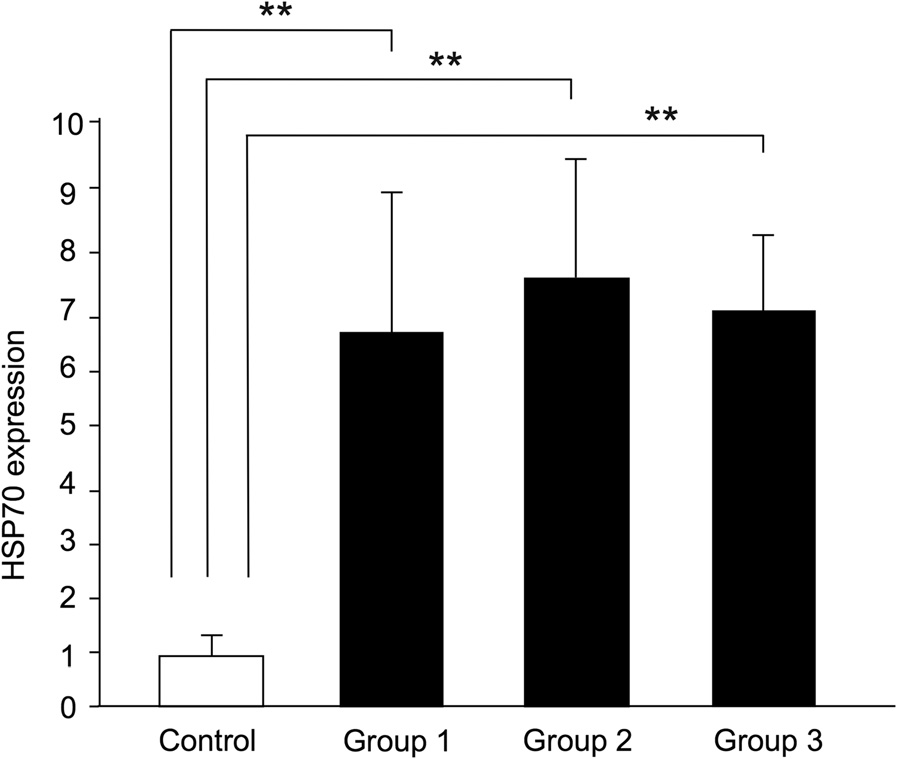
HSP70 mRNA expression at 12 h after the intermittent stimuli 1. The expression of HSP70 mRNA of the control group was significantly lower than the other 3 groups. There was no significant difference among the 3 different mechanical stress groups. The number of times of mechanical stimuli did not show any difference in terms of HSP70 mRNA expression. **p < 0.01

The expression of HSP70 mRNA by the control group was significantly lower than the other 3 groups (*p* < 0.01). There was no significant difference among the 3 different mechanical force groups (Fig. 6). The number of intermittent mechanical stimuli did not show any difference in terms of expression of HSP70 mRNA, which means that once HSP70 mRNA expression occurred, there was no further accumulation of expression.

## Discussion

Although the PDL is continuously exposed to mechanical stresses such as compression forces or extension forces during occlusion and chewing [10–12], those stress responses are considered to represent a cellular defense mechanism against environmental disturbances due to the up-regulated expression of HSPs [17]. These studies demonstrated that ERM cells reduce the functions of hard tissue formation and increase alveolar bone resorption and prevent the PDL from being susceptible to dento-alveolar ankylosis [3, 18].

It has previously been shown that HSPs play an essential role in maintaining the integrity of cytoskeletal structures due to their function as molecular chaperones [9, 22]. Kaarniranta et al. analyzed the effects of hydrostatic pressure (HP) on HSP gene expression by primary human fibroblasts and reported that HSP70 gene expression was detected in human fibroblasts [23]. Their results suggested that HSP70 can play an important role in the early stages of the adaptation of cells to mechanical loading expression. Furthermore, Lui and Kong reported that HSP70 was found transiently upregulated during depolarization and it retained AIF in the cytosol to avoid apoptosis [17].

Lee et al. reported that the addition of intermittent centrifugation to MC3T3-E1 cells stimulated their ALP activity [24]. The expression of HSP70 mRNA at 3 h after centrifugation was the highest and then gradually decreased with time until 36 h in this study. The expression of HSP70 mRNA with intermittent force every 3 h was significantly higher than the control, however, transcriptional level of HSP70 mRNA was not maintained. It is suggested that ERM cells respond to each mechanical force but they have not maintain transcriptional level of HSP70 mRNA in order to protect and support their homeostatic functions.

An advantage to the use of centrifugal force is that it is possible to add even more force to the cells without changing the culture conditions, such as less oxygen or depleted nourishment [13]. Salter et al. demonstrated the integrin as mechanoreceptors that connect components of the extracellular matrix with actin filament in the cytoplasm [25]. Tanaka described the possibility that this pathway via integrin might link the nucleus to the mechanotransduction [26]. SEM observations in this study showed that nuclear protrusions in the control group and flattening of the nuclei occurred immediately after the centrifugation but returned to the original shapes just the same as the control at 3 h after the centrifugation. This suggests that centrifugal force might influence the structural route of actin filaments immediately after the centrifugation, and that the network of signal transmission is activated.

Redlich et al. reported that application of a 1,000 rpm centrifugal force to human PDL fibroblasts promoted cell death by 20 % while the expression of mRNAs encoding type 1 collagen, matrix metalloproteinase and tissue inhibitor of matrix metalloproteinase was increased [19, 20]. Naito et al. reported the effects of 4,800 rpm, which was used in this study and is thought to be similar to the occlusal force and biting strength, on bone marrow stem cells [13]. They concluded that cells subjected to a 4,800 rpm centrifugal force showed a higher cell proliferation activity and expression of bone related protein mRNAs compared to cells centrifuged at 600 rpm.

On the contrary, cells under a cover glass as a compressing force, as used in this study, were reported by Hoshina et al. to potentially become hypoxic and undernourished, but they concluded that 0.9 g/cm^2^ loading by the glass plate had no difference in terms of cellular morphology and functional analysis [14]. They reported that the compressing force might suppress the final cell differentiation in the calcification phase of osteoblastic cells. In this study, the expression of HSP70 mRNA was much higher in the case of the combination of centrifugal and compressing forces than either centrifugal force alone or compressing force alone. These results suggest that the compressing force initially makes changes of the intracellular environment and that the centrifugal force changes the structure of actin filaments and accelerates the change in HSP70 gene expression.

## Conclusions

These findings indicate that ERM cells express HSP70 mRNA in response to mechanical force, and that intermittent force maintains the level of HSP70 mRNA expression. However the expression of HSP70 decreases with time and, transcriptional level of HSP70 mRNA gene was not maintained. For future consideration, analysis of protein is necessary.
